# The Duties of Medical Men to Medical Societies

**Published:** 1887-02

**Authors:** Virgil O. Hardon

**Affiliations:** Lecturer on Operative Gynæcology, Southern Medical College, Atlanta, Ga.


### Southern Medical Record.

A MONTHLY JOURNAL OF PRACTICAL MEDICINE.

☞All Communications must be addressed to R. C. WORD, M. D., Managing Editor.

"*Quicquid Præcipies Esto Brevis.*"

VOL. XVII. ATLANTA, GA., FEBRUARY, 1887. No. 2.

### ORIGINAL AND SELECTED ARTICLES.

### THE DUTIES OF MEDICAL MEN TO MEDICAL SOCIETIES.

*The President's Address, delivered at the Inaugural Meeting of the Atlanta Society of Medicine, January 4, 1887.*

BY VIRGIL O. HARDON, M. D.,
Lecturer on Operative Gynæcology, Southern Medical College, Atlanta, Ga.

*Gentlemen of the Atlanta Society of Medicine :* In assuming the office of President of this Society, to which your preference has elected me, it becomes my duty, in accordance with the requirements of the Constitution, to deliver an inaugural address. Before proceeding to the fulfillment of this task, allow me to say a few words expressive of my high appreciation of the honor which you have conferred upon me—an honor which is all the more grateful from the fact that it comes entirely unsought, and which I may therefore feel warranted in regarding as a flattering evidence of your esteem and confidence.

I speak of the office of President of the Atlanta Society of Medicine as an important one, for such it is. Our city has attained a growth and a prominence which place it among the best known cities of the land. In size it is excelled by only two cities in the South. As a medical centre it is excelled by none. It can boast of two good medical schools, with an aggregate of twenty-eight instructors and over two hundred students drawn from all parts of the country, not excepting even distant New England. It sends out to the professional world two monthly medical journals, with a combined circulation of upwards of five thousand subscribers. These journals are in no sense local publications, since they go to nearly every State in the Union, find many readers in Europe, and even penetrate to such remote regions as Australia and the Cape of Good Hope. No such showing as this can be made by any other city in the South. The distinguishing enterprise and energy of Atlanta are manifested in this respect, no less than in her commercial, financial, mechanical and social preeminence. The extent of the influence exerted by our medical schools and our professional journals can hardly be over-estimated. Our corps of medical teachers embraces men whose fame as physicians and as writers is not limited by the confines of our city, our state or even our country. Our journals give to the profession medical literature of such a character as to be thought worthy of quotation and comment by the leading journals of the world.

It follows that the men who make up the medical profession of such a city are men of whom much may reasonably be expected. We are not a band of provincial, rural doctors, hampered by lack of clinical advantages and opportunities for observation, and dwarfed in our growth by professional isolation and absence of the privileges of intimate association and interchange of thought and experience. It is to us that such men look for encouragement, for instruction, for advice. We cannot shirk this responsibility, if we would. It is forced upon us by the circumstances in which we are placed. In view of our superior facilities for wide experience and observation, the profession has a right to expect from us good work in the advancement of medical science. If we fail to show such good work, then we fail to attain to the full measure of our opportunities, and are derelict in the higher duties which our chosen profession entails upon us.

Unorganized work is, as a rule, inferior work. This principle is as true in the profession of medicine as in politics or religion, in spite of some notable exceptions. The best workers in our profession are found in the ranks of medical societies. This fact is recognized by the profession at large, and is attested by the increased space and prominence given to society reports by the leading medical journals. The medical society is assuming the importance which it deserves by virtue of the superior facilities which it affords for the comparison of clinical facts and therapeutic results; hence it may reasonably be expected that the Atlanta Society of Medicine will act a worthy part in directing medical thought and in adding its little stream to the broad current of progress which is sweeping through the medical science of to-day, leaving stranded upon its banks the worthless *debris* of yesterday, but carrying onward to our successors of to-morrow all that is good and true and worthy of an enduring existence. To direct the work of this Society, to call out the best efforts of each individual member, and to consolidate those efforts for the common good is, therefore, no unimportant office, and no man is warranted in assuming the duties of that office who is not imbued with a deep sense of the responsibilities which thereby devolve upon him.

But it is not upon the President alone that the future of this Society rests. There are duties and responsibilities which you cannot delegate to him. He is but the engineer who stands at the throttle and directs and controls a force of which he is in no wise the originator. If that force is lacking, his courage, his fidelity, his skill will count for nothing. The responsibilities which rest upon each individual member of this Society, if less in degree, are in no wise less urgent than those which rest upon your presiding officer. It is in pursuance of this line of thought that I wish to ask your attention to what I shall have to say upon "The Duties of Medical Men to Medical Societies."

The highest ideal type of a physician is that of a man who is fitted by nature and by education for the duties of his profession—whose whole heart and soul are wrapped up in the alleviation of human suffering, and who, in the pursuance of this object, gives no thought to private interest, personal comfort, or pecuniary reward. But this ideal is never fully realized, though some approach it. The exigencies of modern life require that every man should "take thought for the morrow." The doctor must dress, must eat and drink, must rear and educate his children ; he cannot, therefore, entirely ignore the pecuniary side of his work. A due regard for those whose welfare and happiness are dependent upon his life and health, demands that he should take such recreation and rest as will prevent the shortening of his life from overwork ; hence he must divert his mind by other pursuits in whatever direction may chance to accord with his tastes. The ideal physician, therefore, like most other ideals, can never find its realization in actual life. A greater or less degree of approximation to it is all that can be hoped for.

From the standpoint of this ideal there exist two classes of physicians. The one class includes those men who regard the practice of medicine simply as a means of getting money. These men are so busy with their little muck-rakes that they have no time to look up from their sordid occupation to recognize the existence of higher and better things. For such men my remarks are not intended. To speak to them of professional duties would be like "casting pearls before swine." But, fortunately, there is another class of practitioners to whom the practice of medicine is a profession, and not a trade ; who recognize the element of humanity and of morality involved in that profession, and who are constantly striving, by earnest, conscientious endeavor, to approximate to the ideal of the true physician. It is to such men that my remarks are addressed, and it is by such men alone that my views will be appreciated.

To this latter class of men—which I am proud to believe includes the larger part of the profession—two distinct lines of duty present themselves. The first is the plain, unmistakable duty which the physician owes to his *clientele.* No honest man can practice medicine with a clear conscience who has not availed himself of every opportunity within his reach to fit himself for rendering to those who seek his aid the best professional service that the science of medicine can afford. I know of no more harrowing thought that can haunt a man than that a human life has been lost by his wilful ignorance or incompetence. The public has no choice but to place a blind confidence in a man who offers to them his medical services, since there is no means by which the competent can be distinguished from the incompetent. To abuse this confidence is to betray a sacred trust and to tamper with human life for the sake of sordid gain. It is the duty, therefore, of every physician, which he owes to his patients, to justify their confidence in his ability and skill by fitting himself to the utmost limit of his opportunities for the prevention and cure of disease.

But there is another line of duty which must present itself to the thoughtful and conscientious physician, and which, though less obvious and immediate than his duty to his patients, is none the less imperative. I refer to the duty which he owes to those who shall come after him. The profession of to-day has received a valuable foundation of medical knowledge from its immediate predecessors upon which to build a still more lofty superstructure. To our fathers we owe a debt which we can never repay to them in person ; but we can cancel the obligation thus incurred by striving to transmit to our successors of to-morrow all that we have received, with a rich increase. A belief in the brotherhood of man makes it no less incumbent upon us to work for those who shall come after us than to work for those who are our immediate contemporaries. He who saves from death to-day a fruitful woman will have made an addition to the sum total of existence in the ages yet to come, which will go on ever increasing and widening out to the end of time ; and so, he who establishes one fact or formulates one principle upon which can be founded a more successful treatment of disease will, as the years go by, have relieved an incalculably greater amount of human suffering than he who devotes his energies solely to the benefit of the afflicted of his own day and generation. Thus when Marion Sims achieved immortality by giving to the world the speculum which bears his name, he conferred upon our race a benefit which shall be limited only by the duration of the solid earth itself. Which, then, is the greater duty, to heal the individual of to-day, or to heal the race of the future ? Or, rather, is it not true that both alike demand the attention of the conscientious physician ?

To the practitioner of medicine who has finished his collegiate course and entered upon his professional career, three methods offer themselves by which he may increase his store of medical knowledge : they are personal observation, study of medical literature, and discussion and comparison of views with fellow-practitioners. All are alike valuable and, if properly pursued, are productive of good results. Passing over the first two methods as not germane to the subject of this address, and attempting no comparison of the relative value of the three, I shall attempt to show that the last offers to the medical man advantages which can be gained in no other way.

The tendency of the medical mind when isolated from contact with other minds is to run into one or the other of two extremes : either to degenerate into blind, unreasoning routine or to soar into the realms of fanciful transcendentalism. There are very few men, in ours or in any other profession, who have an intellect which, when they are left to themselves, is sufficiently well balanced to prevent their falling into one or the other of these dangers. The one results in the production of the intellectual myope who knows nothing beyond the narrow range of his own near-sighted vision ; the other produces the reverse of this—the man whose hypermetropic mental sight can appreciate only the distant abstract. Both are equally unfit for good professional or scientific work. To combat these tendencies no agency is so effectual as the free discussion and comparison of individual opinions and experiences. When brought into contact with other minds, the routinist is surprised to learn that there are facts entirely outside the range of his narrow experience ; that there are useful remedies other than those which make up the contents of his saddle-bags or medicine-case ; that his few meagre generalizations, based upon his limited experience, when brought into contact with similar generalizations of other men, may combine to solve, as neither could do alone, complex questions of pathology or treatment of untold value in medical practice. In the same way the transcendentalist finds, to his chagrin, that when subjected to the strong light of hard common-sense and unbiased criticism, his theories will often evaporate into thin air ; he discovers that one well-attested fact is of greater value than an hundred unsupported hypotheses, and that the principles which underlie the treatment of disease cannot be evolved from the depths of his own consciousness. Thus the two extremes, with the innumerable intermediate gradations, are brought to a happy medium which represents the line along which alone can true progress in medical science be effected. As so uncertain a thing as the expectation of human life may be reduced to an unvarying average by the aggregation of a sufficient number of lives, so, by combining the fruits of many individual experiences and modes of thought, a result is reached which approximates much more closely to the correct solution of a given question than can any individual experience or opinion. Individuality must be lost sight of, the personal equation must be eliminated, before a scientific principle can represent absolute truth.

Herein lies the work of the Medical Society—to bring together the plodder, the listener, the observer, the reader, the writer, the talker, the theorizer, the man of many ideas, the man of one idea, the man, perchance, of no ideas at all, whose mind is a mere machine ; to draw out the best efforts of each in his own peculiar phase of thought ; to contribute to the development of such attributes as each may feel himself most to lack ; to grind down by mental attrition the salient angles and sharp lines which present themselves too prominently ; and, having accomplished these results, to so consolidate the harmonious work which is thereby rendered possible, that each may appropriate his share of the common result and become better fitted for his daily duties to suffering humanity. That such results are possible is proven in a measure by the history of this Society, short though that history is. There is no one who has watched, as I have watched, the working of this Society during the past two years, and its effects upon those men who have availed themselves of its benefits, but will bear me out in the assertion that it has accomplished in a greater or less degree each of the good results which I have named. If, then, it be the duty, as it unquestionably is, of every practitioner to seize every opportunity to fit himself more fully to meet the requirements of his profession, and if, as I feel warranted in claiming, the medical society furnishes such advantages to this end as cannot be otherwise obtained, it is obvious that a solemn obligation rests upon every medical man to associate himself with such organizations. And this constitutes what I consider the first duty of medical men to medical societies.

Involved in this duty, and growing out of it, is the obligation resting on every man to aid in the work of the society according to the measure of his ability. In order that each may be benefited all must contribute to the common stock. The experiences and views of one man upon any subject are of little value until they are tempered and modified and moulded by the opinions of other men. Hence, in order to accomplish good work in the society, it is necessary that each should contribute whatever is furnished by his own experience and observation. There is no man whose field is so limited that he cannot extract from it something which will be of value to his fellow-practitioners. It is not necessary to be a Gross, a Spencer Wells, or a Ricord in order to be an observer. The report of a successful ovariotomy is of far less value than the report of a successful method of treating infantile diarrhœa. The most commonplace and every-day diseases furnish abundant field for observation and study. Who, for instance, can explain the true pathology of whooping-cough or tell how to treat it satisfactorily? He who shall accomplish this will do a far greater good to his fellow-beings than he who performs an hundred consecutive laparotomies without a death. The mortality from whooping-cough is infinitely greater than from salpingitis or cystic ovary. The proper field for study lies immediately before us, and we need not go out of our way to seek it. If every member of this Society would lay out some one line of observation and pursue it patiently, thoroughly and conscientiously—collecting, recording and analyzing cases, comparing the results of such observation with the results obtained by other laborers in the same field, then bringing the fruits of such research before this Society and publishing them in its proceedings—if every member would earnestly adopt this plan, in five years the Atlanta Society of Medicine would accomplish an amount of valuable work which would make its name known throughout the length and breadth of the land. When we consider that the whole science of medicine as it exists to-day is made up of the aggregation of such individual research, and that whatever growth and progress that science shall make in the future must be brought about in the same way, it seems to me that our duty, as a part of the medical profession, to aid in the carrying on of this work is an unmistakable one. And this constitutes what I consider the second duty of medical men to medical societies.

Besides the duty which the physician owes to his *clientele* and to the profession, there is also the duty which he owes to his successors. It is not enough that the results of his labors should be given to those with whom he is brought into contact in the medical society. If his work is of any value to his fellow-members, it is of equal value to the profession at large, and should become a part of medical literature to be handed down to future generations. If it possesses any merit it will find its place, and have its weight, in the solution of vexed problems. We cannot determine beforehand the value of our work. A vast machine may lie idle for lack of a single bolt or pinion ; and so a single fact deduced by one of us from our own study and observation, if given to the world, may prove to be the missing part which is alone lacking in the perfection of some great truth.

Perhaps it will be said that we cannot hope to produce anything which will be worthy of perpetuation. This is not the plea of modesty ; it is the plea of indolence. The history of the past contradicts such a statement. J. Marion Sims, an unknown man, practicing in the little city of Montgomery, Ala., became the father of modern gynæcology ; Ephraim McDowell, from the wilds of a frontier State, gave to the world ovariotomy ; Crawford W. Long, an obscure country doctor of Jackson County, Ga., was the first to use ether for the induction of anæsthesia ; Robert Battey, amid the mountains of North Georgia, patiently wrought out and courageously applied the principles underlying the operation which bears his name. Here were four Southern men, far away from the medical centres, far away from the great medical schools and the great hospitals, who effected discoveries each of which forms an era in the history of medicine. Are our opportunities less than theirs ? In the light of such striking examples, is it unreasonable to hope that patient, well-directed labor might produce a Sims, a McDowell, a Long or a Battey from our ranks ? But such results are achieved not by inspiration or by accident, but by patient, unremitting toil. The men whom I have named were close observers and close thinkers, qualities which, in a large measure, come by cultivation. The acquirement of the habits of close observation and correct reasoning is one of the results of society work. The proceedings of medical societies which appear in our journals represent the most advanced thought of the day. The most valuable articles given to the profession are those which have first been read in the medical societies. Such work should not be lost in verbal reports or desultory, off-hand discussion which restrict its benefits to the immediate listeners. It is the boast of our profession that it is free from all exclusiveness—that what is the property of one is the common property of all. In accordance with this principle it is the duty of every man who establishes a new fact or formulates a new theory, to subject it to the criticism of his fellow-practitioners, and, if it survives this ordeal, to give it to the profession at large that it may find its true place and play its peculiar part in the advancement of medical science. And this constitutes what I consider the third and final duty of medical men to medical societies.

Such, then, are the duties which rest upon us as practitioners of medicine. They are not matters of privilege or of choice, but of solemn obligation. By him who regards the healing of his fellow-men as a lofty and noble profession, these responsibilities cannot be ignored. It is to the earnest and thoughtful consideration of such men that I offer the views presented in this address.

